# Rapid assembly of colorless antimicrobial and anti-odor coatings from polyphenols and silver

**DOI:** 10.1038/s41598-022-05553-9

**Published:** 2022-02-08

**Authors:** Joseph J. Richardson, Wenting Liao, Jincai Li, Bohan Cheng, Chenyu Wang, Taku Maruyama, Blaise L. Tardy, Junling Guo, Lingyun Zhao, Wanping Aw, Hirotaka Ejima

**Affiliations:** 1 Department of Materials Engineering, School of Engineering, University of Tokyo, Tokyo, 113−8656 Japan; 2 Department of Bioproducts and Biosystems, School of Chemical Engineering, Aalto University, 02150 Espoo, Finland; 3 BMI Center for Biomass Materials and Nanointerfaces, College of Biomass Science and Engineering, Sichuan University, Chengdu, 610065 Sichuan China; 4 State Key Laboratory of Polymer Materials Engineering, Sichuan University, Chengdu, 610065 Sichuan China; 5 State Key Laboratory of New Ceramics and Fine Processing, School of Materials Science and Engineering, Tsinghua University, Beijing, 100084 China; 6 Institute for Advanced Biosciences, Keio University, 246-2 Mizukami, Kakuganji, Tsuruoka, Yamagata 997-0052 Japan; 7 JST-PRESTO, Honcho 4-1-8, Kawaguchi, Saitama 332-0012 Japan

**Keywords:** Organic-inorganic nanostructures, Nanoscale materials, Antimicrobials

## Abstract

The development of antimicrobial fabrics and textiles that can sustainably inhibit a broad spectrum of microbes is crucial for protecting against pathogens in various environments. However, engineering antimicrobial textiles is challenging due to issues with discoloration and inhibited breathability, the use of harmful or harsh reagents and synthesis conditions, and complex and/or time-consuming processing. Herein, we develop a facile and rapid approach to deposit antimicrobial coatings using universally adherent plant polyphenols and antimicrobial silver ions. Importantly, the coatings are colorless, thin (< 10 nm), rapidly assembled (< 20 min), and can be deposited via immersion or spraying. We demonstrate that these metal-phenolic coatings on textiles can inhibit lipid-enveloped viruses over one thousand times more efficiently than coatings composed of other metal ions, while maintaining their efficacy even after 5 washes. Moreover, the coatings also inhibit Gram positive and negative bacteria, and fungi, and can prevent odors on clothes for at least 10 washes. Collectively, the ease of synthesis, use of simple and safe precursors, and amenability to at-home and industrial application suggests that the coatings will find practical application in various settings.

## Introduction

Antimicrobial materials have played an important role in modern society by preventing and treating diseases, and boosting quality of life^1^. Antimicrobial coatings are a specific class of antimicrobial materials that can be deposited onto substrates to either neutralize microbes or prevent them from interacting with the substrate, and a wide range of coatings have been developed to date^2–5^. However, it is challenging to readily engineer antimicrobial coatings for soft and porous substrates, such as natural and synthetic textiles, without negatively impacting the textile. For example, many antimicrobial coatings require complex strategies to apply the coatings and have undesirable qualitative properties that can limit their use, such as heavy leaching during use or washing, high absorbance of visible light and corresponding discoloration, clogging of textile pores, or the incorporation of potentially toxic compounds^5–10^. Therefore, it is important to develop colorless antimicrobial coatings from safe reagents that can rapidly be deposited on various textiles in ambient conditions and that last multiple washes.

**Scheme 1:**
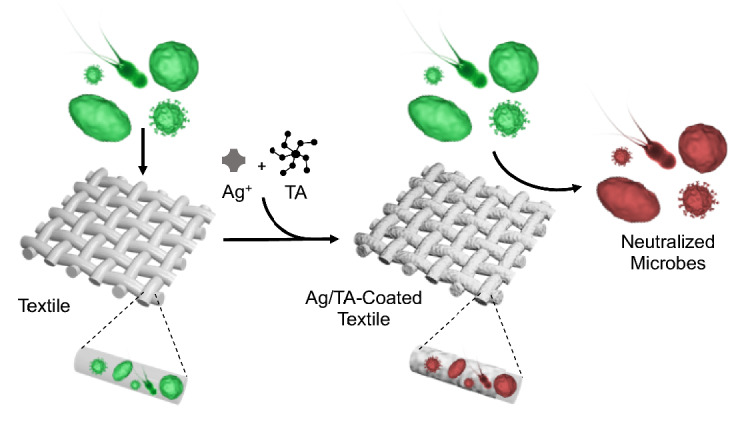
Schematic illustration of the formation of colorless Ag/TA coating on textiles and their interactions with microbes. While pathogenic and odor-causing microbes can survive on uncoated textiles, Ag/TA-coated textiles rapidly neutralize a wide range of microbes.

Metal-phenolic networks (MPNs) are an emerging class of amorphous coordination polymers that can be deposited on nearly any substrate in ambient aqueous conditions due to the versatile chemical nature of catechol and gallol groups^11^. The synthesis process is rather robust to concentrations, phenolic ligand choice, and metal ion choice, and a library of functional coatings, generally on the order of 10 nm, have been generated to date for various applications^11–16^. Previously, MPN coatings composed of tannic acid (TA) and Fe(III) have been used as a barrier to minimize the adhesion of microbes to substrates and separately, to form Ag nanoparticles in reducing conditions to provide antimicrobial benefits, however both of these approaches resulted in significant discoloration and were not tested for resistance to washing, while the latter additionally required a multi-step synthesis process spanning roughly 1 day^5,17^. Herein, we demonstrate that colorless metal-phenolic coatings utilizing Ag^+^ can provide antimicrobial benefits to a range of textiles (e.g. cotton, synthetic fiber, silk) (Scheme 1). Specifically, the colorless coatings composed of Ag(I) and TA (Ag/TA) could easily and rapidly (under 20 min) be applied via immersion or spraying onto complex substrates including textiles. These coatings inhibited lipid enveloped viruses (phi6), Gram negative and positive bacteria (E. coli and S. aureus, respectively), and fungi (S. cerevisiae), and could neutralize odor-causing bacteria on clothes in real-world application for at least ten wear/wash cycles. The beneficial properties of these coatings in combination with their non-toxic building blocks suggests they could find use in various settings such as hospitals, food-processing plants, and sports complexes.

## Results and discussion

**Figure 1:**
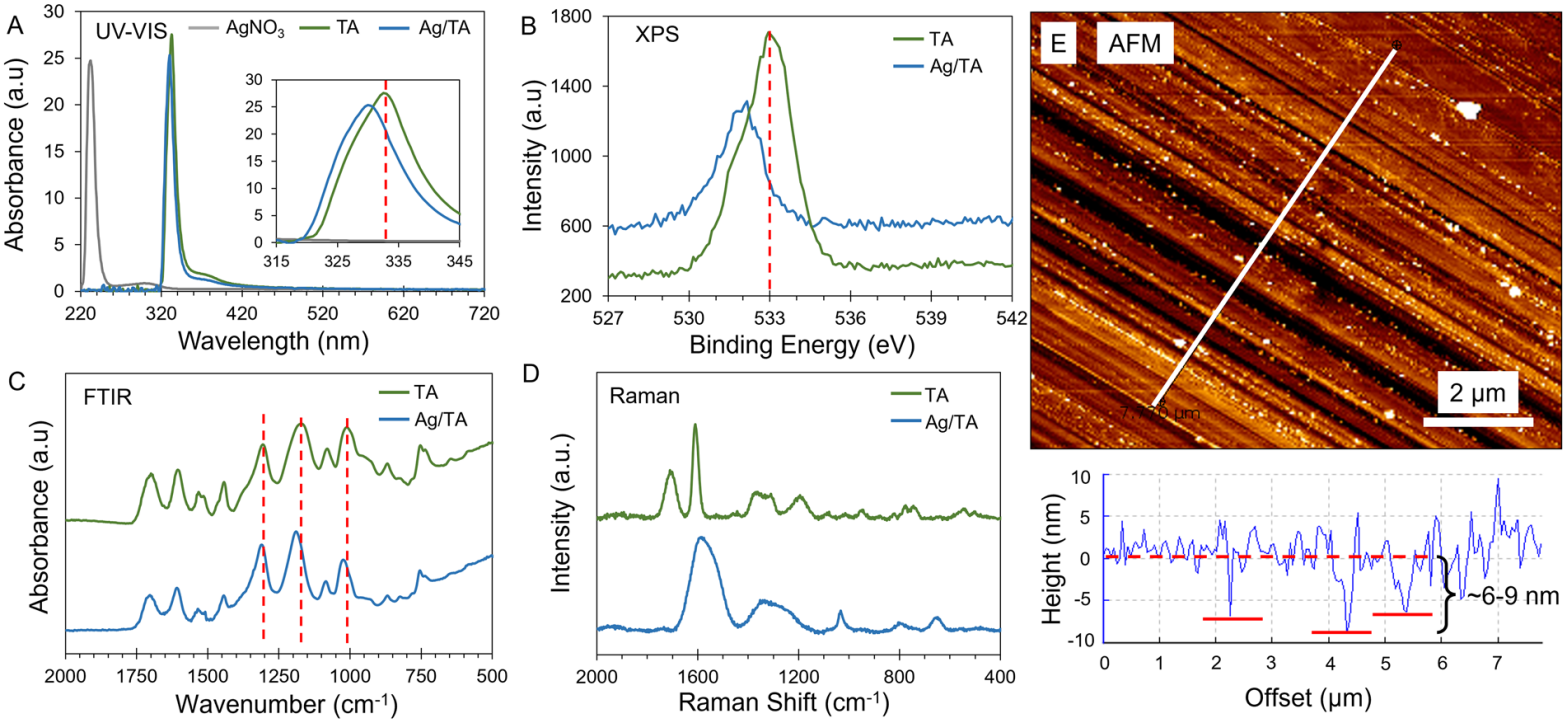
Ag/TA complexes and coatings. (**A**) UV–VIS spectra of aqueous solutions of AgNO_3_, TA, and Ag/TA complexes. XPS (**B**), FTIR (**C**), and Raman (**D**) spectra of TA and Ag/TA complexes. (**E**) AFM image and height profile of a scratched Ag/TA coating on silicon deposited in ~ 10 s. The red dotted lines in (**B**) and (**C**) represent peaks of TA where shifts are seen for Ag/TA. The red dotted line in (**E**) represents the average height of the film, with the solid red lines signifying the underlying silicon wafer after scratching.

The Ag/TA coatings were deposited in ambient aqueous conditions similar to other metal-phenolic coatings^11^. Generally, the substrate was first immersed in 500 µL of ultra-pure water followed by the addition of 5 µL of 40 mg mL^−1^ phenolic solution (TA, or other phenolics) and subsequent mixing. Next, 5 µL to 60 µL of 10 mg mL^−1^ AgNO_3_ solution was added followed by vigorous mixing and incubation. The coatings were then rinsed with ultrapure water, dried by air or nitrogen, and then used for subsequent studies. After the addition of AgNO_3_, complexation rapidly occurred as seen by a near immediate (< 10 s) size increase in dynamic light scattering results from ~ 2 nm (roughly the radius of gyration of TA) to ~ 100 nm (a similar size to Fe/TA complexes seen in solution) (Fig. S1)^14^. Moreover, the silver peak in UV–Vis spectroscopy disappeared after complexation and the TA phenolic peak shifted to slightly lower wavelengths and no new peaks appeared, suggesting that films made out of these complexes would be transparent (Fig. 1A). X-ray Photoelectron Spectroscopy (XPS) demonstrated shifts in the C_1s_ spectra of TA to lower binding energies after interacting with Ag (from 533.1 eV for TA to 531.8 eV for Ag/TA complexes) and noticeable shifts to higher binding energies in the Ag_3d_ spectra for the two silver peaks (from 374.1 eV for AgNO_3_ to 374.5 eV for Ag/TA complexes) (Figs. 1B and S2)^18^. Fourier-transform infrared (FTIR) spectroscopy demonstrated a shift in the C–O peaks of TA (1011, 1070, and 1300 cm^−1^) to higher wavenumbers (1023, 1189, and 1303 cm^−1^, respectively), which is a signature of chelation (Fig. 1C)^19,20^. Similarly, Raman spectroscopy demonstrated increased peak intensity of TA after interacting with Ag, which is common for metal chelation with phenolic compounds (e.g. catechol-metal binding peak at 649 cm^−1^) (Fig. 1D)^21,22^.

**Figure 2:**
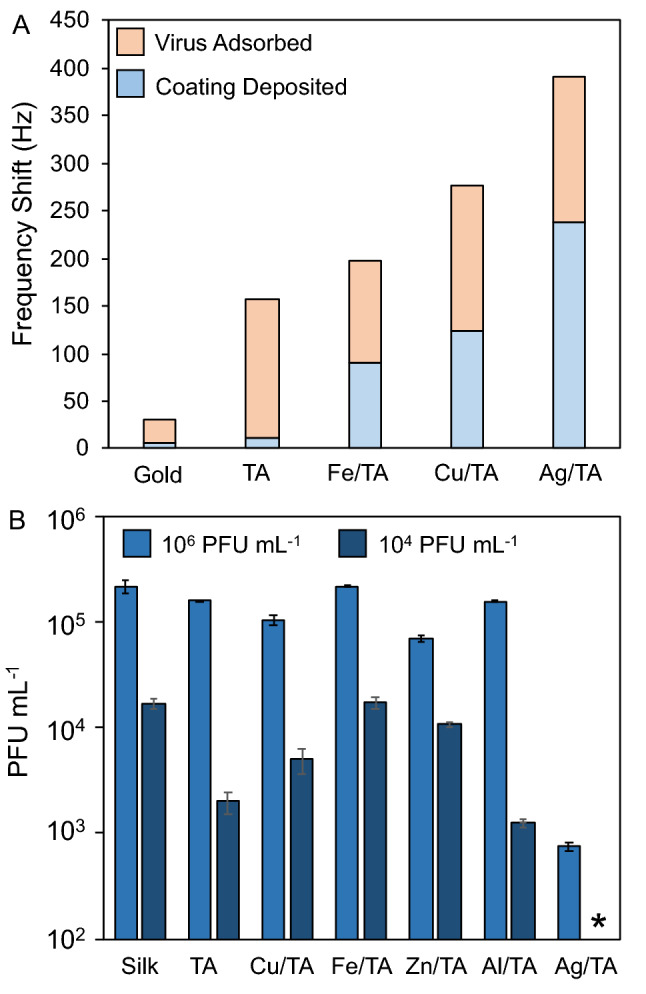
Metal/TA coatings and their ability to interact with and neutralize viruses (phi6). (**A**) Frequency shift (1st overtone) and associated mass of coatings deposited using TA by itself and with different metals and their subsequent ability to interact with viruses as monitored by QCM. (**B**) Antiviral properties of coatings on silk made from TA by itself and with different metals at different virus concentrations. Note that no virus was detectable at the lowest concentration (10^4^ PFU mL^−1^) when incubated with Ag/TA (marked with *), where 10^2^ PFU mL^−1^ was the minimum detectable concentration of the assay.

Importantly, the coatings made from Ag/TA had negligible absorbance in the visible light spectrum (i.e., were colorless as seen by eye) even after 2 h incubation, with a broad peak in the UV spectrum (Figs. S3 and S4). Note that the pH was not raised after mixing to maintain the transparency of the coatings as both NaOH and buffers are known to induce the formation of Ag nanoparticles, which have a strong unappealing color in solution and on substrates (Fig. S4). Atomic force microscopy (AFM) images of scratched coatings showed thicknesses of roughly 6–9 nm with root-mean squared roughness of ~ 1.6 nm after 10 s of deposition, with only a slight increase in thickness and roughness even after 20 min incubation (~ 10 nm and ~ 2.7 nm RMS roughness), which are both comparable to metal-phenolic coatings made from other metals (Figs. 1E and S5)^11,12^. The thin nature of Ag/TA coatings suggests that coated textiles will remain breathable. The Ag/TA coatings exhibited over an order of magnitude more mass than a monolayer coating of TA as measured by quartz crystal microgravimetry (QCM) (Fig. 2). Increasing concentrations of Ag (higher ratio of Ag to TA) led to coatings with more adsorbed mass up to a saturation point after which the mass deposited was fairly stable, likely due to the increased amounts of Ag in the resultant coatings until the free binding sites of TA were fully saturated (Fig. S6).

We recently demonstrated that MPN coatings can interact with lipids, proteins, and polysaccharides^11,16,23,24^, suggesting they will be able to interact with the envelopes, capsids, cell membranes and cell walls of viruses, Gram negative and positive bacteria, and fungi. Therefore, the performance of the Ag/TA coatings to capture and neutralize lipid-enveloped viruses was first compared against coatings with different metals using phi6, a safe-to-study bacteriophage often used as a model for pathogens such as Zika and SARS-CoV-2^25,26^. The Ag/TA coatings could adsorb a similar amount of virus to TA monolayers and Cu/TA coatings, and roughly 50% more virus mass than Fe/TA coatings (Fig. 2A). In terms of virus neutralization, Ag/TA outperformed coatings made from TA alone and outperformed metal-phenolic coatings made from other metals (Cu, Zn, Al, Fe) and TA with a 3-log to 4-log reduction at lower virus concentrations (10^4^ plaque forming units (PFU) mL^−1^) and 2-log to 3-log reduction at higher virus concentrations (10^6^ PFU mL^−1^) (Fig. 2B). Note that Phi6 was only detectable after incubation with Ag/TA-coated materials at concentrations above 10^4^ PFU mL^−1^ as the detection limit of the assay was ~ 10^2^ PFU mL^−1^. After removing bound virus with surfactant from the textiles incubated at high virus concentrations, their viability was checked and again Ag/TA had at least 2-log fewer viable virus than all of the other samples (Fig. S7). Importantly, the antiviral performance of the Ag/TA coatings was maintained for at least 5 vigorous soap and water washing steps, suggesting that such coatings could be used for continued antiviral protection in everyday settings (Fig. S8). Finally, other ligands, such as persimmon tannin, could also be used to form Ag/phenolic coatings with comparable antiviral performance to Ag/TA, demonstrating the versatility of this approach (Fig. S9).

The ability to utilize other phenolic ligands, in addition to the clear superiority of Ag in comparison to other metals, suggested that the specific metal was more important than the specific phenolic ligand in terms of antimicrobial efficacy. The antimicrobial mechanism of silver is well-studied and generally Ag^+^ is considered the most potent form of silver, with Ag nanoparticles generally demonstrating efficacy due to their release of Ag^+^ in the presence of oxygen dissolved in water or in air^27,28^. The antimicrobial effects of Ag^+^ arise from multiple synergistic factors including membrane disruption and DNA damage via reactive oxygen species generation^29,30^. Materials capable of releasing Ag^+^, such as films containing Ag nanoparticles, have found use for antimicrobial applications^5,31,32^, however only recently has the incorporation of Ag(I) into functional materials received interest^33,34^, potentially due to the challenge of stabilizing Ag(I) in complex environments. Interestingly, chelated Ag(I) is significantly more potent than free Ag^+^^35^, possibly explaining why Ag/TA has such a high antiviral efficacy in comparison to the other metals tested. Therefore, we suspected that the Ag/TA coatings could be applied to the inhibition of other microorganisms.

**Figure 3:**
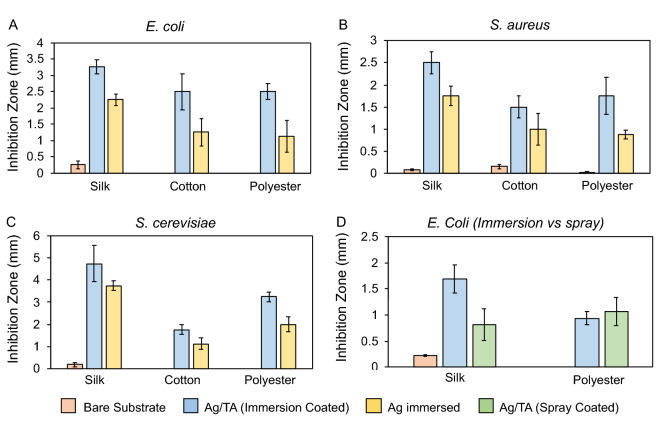
Antibacterial and antifungal properties of Ag/TA coatings on different textiles. Inhibition zones of uncoated, Ag/TA-coated, and AgNO_3_-immersed silk, cotton, and polyester after incubation with *E. coli* (**A**), *S. aureus* (**B**), and *S. cerevisiae* (**C**). (**D**) Comparison of *E. coli* inhibition between Ag/TA coatings applied with immersion or spraying on silk and polyester.

Although viruses are topically relevant due to the ongoing COVID-19 pandemic, bacteria and fungi pose constant threats to health and quality of life^36^, and therefore the Ag/TA coatings were tested against these microbes using different textiles (i.e., cotton, silk, and polyester). The Ag/TA coatings showed significant inhibition on all textiles to *E. coli*, *S. aureus*, and *S. cerevisiae*, while generally the uncoated textiles showed negligible inhibition to these three microbes (Fig. 3). Textiles soaked in AgNO_3_ showed partial inhibition, while uncoated silk also showed some partial inhibition in certain scenarios, as previously documented^37^. Moreover, after incubation with *E. coli*, the Ag/TA-coated textile and uncoated textile were left to sit in LB growth media overnight, and the OD600 of the uncoated textile (high turbidity) was at least 2-log higher than that of the Ag/TA-coated textile (transparent), with OD600 values of 26.6 vs 0.1 for paper, 2.21 vs 0.01 for silk, and 27.3 vs 0.27 for polyester, respectively (Fig. S10). Notably, textiles coated with Ag/TA via spraying (rather than immersion), also showed inhibition zones, though they were slightly smaller likely due to the lesser amount of silver available in the small volumes used for spraying when compared to the far excess volumes used in immersion (Fig. 3D).

**Figure 4:**
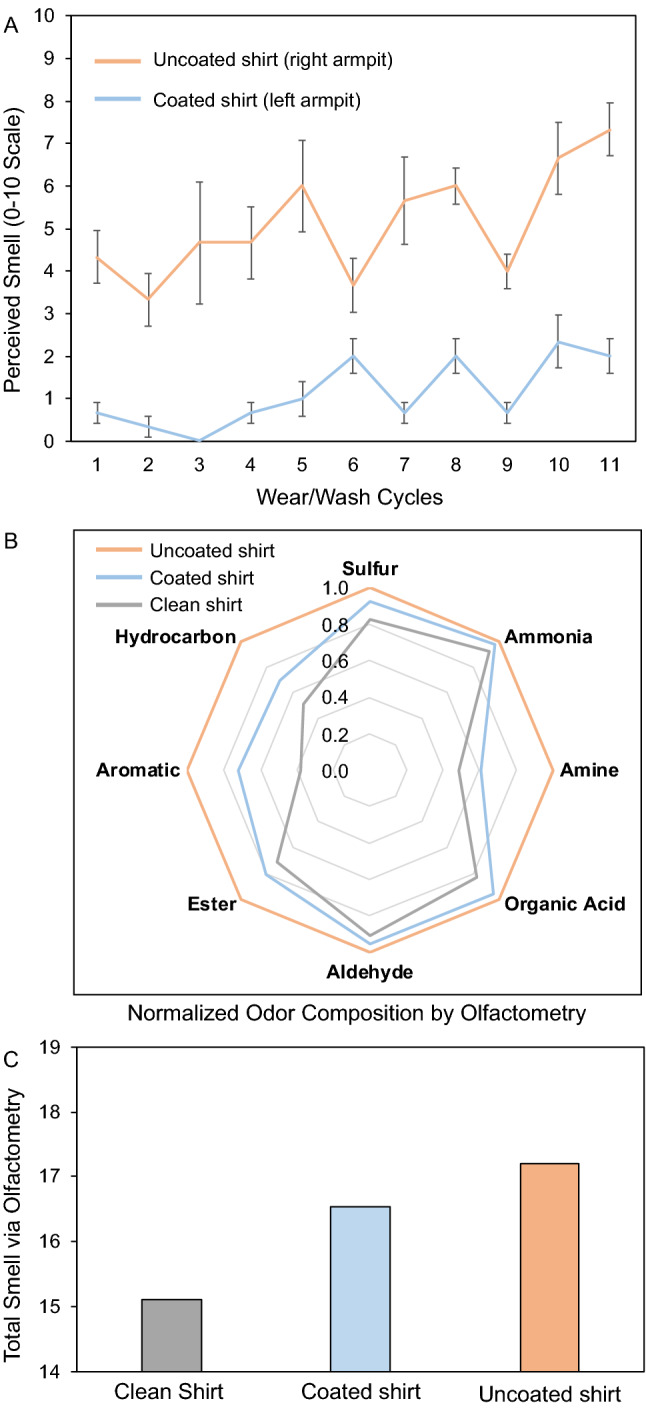
Odor prevention of Ag/TA coatings on clothes after a full day of wear. (**A**) Coatings were only applied once, and then the clothes were worn normally, and machine washed and dried normally. Note the average daily temperature increased over the lifetime of the experiment. (**B**) Normalized odor composition of fabric from the same shirt before wear (clean shirt) and after wearing (Ag/TA-coated left armpit and uncoated right armpit) as measured by olfactometry. Note that the shirt was composed of synthetic fibers, was purchased new and washed once before collecting the samples. (**C**) Total strength of the overall smell from each sample in (**B**).

Microbes can impact human health, but also play an important role in other biological process such as body odor^38,39^. Therefore, we further tested whether fabrics sprayed with Ag/TA coatings could reduce odor caused by normal daily life. By coating only one armpit of shirts (polyester and cotton), internal controls between coated (left) and uncoated (right) fabric could be maintained on a day-to-day basis by comparing the smell on a 0 to 10 scale at the end of the day (after 10–12 h of wear). Roughly 350 µL of each precursor was sprayed sequentially onto the armpit (roughly 7 × 7 cm) of the shirts and left to dry, afterwards the shirt was worn and machine washed normally. Notably, the Ag/TA-coated armpits were nearly odorless across 10 wear/wash cycles, and showed significantly less odor than the uncoated armpits in all scenarios (Figs. 4 and S11). Specifically, the uncoated armpit averaged a pungent smell of 5.3 over 3 trials of 11 wears and 10 washes each, while the Ag/TA-coated armpit has a nearly imperceptible average smell of 1.2 over these same wears. The ability of the coatings to withstand machine washing with commercial detergent was expected as phenolic molecules in food and drink can cause stains (e.g., chocolate, wine, and coffee stains) that are notoriously difficult to remove^40^. When analyzed by an olfactometer, the difference between the Ag/TA-coated and uncoated shirt armpits was also obvious (Fig. 4b). Notably, the Ag/TA-coated armpit was more similar in odor profile to the control clothing that had never been worn, than it was to the uncoated armpit (Fig. S12). This ability to neutralize odor-causers, in combination to the colorless nature of the coatings, suggests that Ag/TA will be able to find use in various niches of everyday life.

## Experimental

### Deposition of metal-phenolic coatings on different substrates

Metal salts were dissolved at 10 mg mL^−1^ and phenolics were dissolved at 40 mg mL^−1^. Generally, the substrate was first immersed in 500 µL of ultra-pure water followed by the addition of 5 µL of 40 mg mL^−1^ phenolic solution (TA, or other phenolics) followed by mixing. Next, 5 µL to 60 µL of 10 mg mL^−1^ metal solution (e.g., AgNO_3_ solution) was added followed by vigorous mixing and incubation. To alter the ratio, different volumes of each precursor was used. The substrates were then rinsed with ultra-pure water and generally air dried before use. Further washing with soap and water was performed for some experiments to test the robustness of the coatings.

### Phi6 culturing

Phi6 (NITE Biological Resource Center (NBRC), #105899) was cultured using the host organism, *P. syringae* (NBRC #14084) in culture media (10 g hipolypeptone, 2 g yeast extract, and 1 g MgSO_4_ in 1 L water). Briefly, *P. syringae* was cultured overnight at 25 °C, followed by the addition of Phi6 (1 mL in 250 mL) and incubated for 4 h, followed by pelleting at 5000 rcf. The supernatant containing the virus as the filtered through a 0.22 µm syringe filter. This was then stored at 4 °C in the dark. Phage buffer (100 mM NaCl, 8 mM MgSO_4_ and 50 mM Tris hydrochloride) was used when necessary to dilute Phi6. The concentration of Phi6 was then determined using plaque assays (described below) with serial dilutions.

### QCM study of virus adsorption

Phenolic and metal-phenolic coatings were applied to Au-coated QCM chips (9 MHz, Seiko EG&G), followed by rinsing with deionized water and drying with nitrogen. The quantities of phenolic and metal-phenolic coatings adsorbed on the surface were determined according to the frequency change (QCM-992A, SEIKO EG&G). Next, the coated and uncoated QCM chips were incubated in a vial of 1 mL of Phi6 (10^6^ PFU mL^−1^) for 10 min. After incubation, the chips were rinsed with deionized water and dried with nitrogen before measurement of the frequency change.

### Virus inhibition assays

The drop method was used for testing the inhibition of Phi6. Briefly, an overnight culture of *P. syringae* was added to fresh media and cultured overnight again. 10 µL of virus solution at the desired PFU mL^−1^ was added to 90 µL of growth media and serially diluted at least three times. 10 µL of bacteria was added to each of these samples, and the tubes were incubated at 30 °C for 20 min. Finally, 20 µL of each sample was dropped in duplicate on agar plates of the growth media. This was left to dry in the biosafety hood, and then cultured overnight at 25 °C upside down. The plaques were then counted and the PFU mL^−1^ was calculated by multiplying the number of plaques by 50 and the dilution factor.

### Bacteria and fungi experiments

*Escherichia coli* (NBRC# 3972, cultured in LB), *S. aureus* (NBRC# 12732 in LB), and *S. cerevisiae* (NBRC# 10217 in YPM) were cultured normally according to the supplier’s protocol. Antimicrobial tests were performed by adding 100 µL of overnight cultures of the microbes to an agar plate, followed by uniform spreading and subsequent drying. The substrates were then placed on the plates, and cultured inverted overnight. Inhibition zones were then measured with a ruler.

### Spray deposition and odor protection experiments

Solutions of 0.1 mg mL^−1^ AgNO_3_ and 0.4 mg mL^−1^ TA were sequentially sprayed onto substrates from a distance of ~ 10 cm. Generally, 10 µL of each precursor was sprayed per cm^2^ with an average spray volume of 30 µL. After coating, the substrates were then washed and dried normally. For the odor protection experiments, the left armpits of different shirts were coated, while the right was kept uncoated as a negative control. After a day of wear (generally including a ~ 50 min of roundtrip biking to and from work) by a Caucasian male between the age of 20 and 45, the shirt was removed and each armpit was smelled at ~ 1 mm distance from the nostrils. The odor was rated on a 0 to 10 scale for each armpit and the experiments were conducted from May to August. Between wears, the clothes were machine washed normally using detergent and tumbled dry with normal heat.

For the olfactometry experiments, a new shirt was washed and then a portion of the shirt was cut off for reference. The left armpit was then coated with Ag/TA and the right was left uncoated and the same male then wore the shirt from 8 A.M. to 8 P.M. including ~ 90 min of hiking the day before analysis. After removing the shirt, the armpits were cut out of the shirt and separately sealed in bags, as was the reference fabric. The next day the samples were separately incubated for 2 h in N_2_ gas, after which the gas from each sample was transferred to an analysis bag, and analyzed on a Shimadzu FF2020.

## Conclusions

In conclusion, we report the fabrication of transparent Ag/TA coatings for textiles that demonstrate antimicrobial and anti-odor properties over multiple washes. This strategy adds to the versatile toolbox of MPNs and metal-phenolic coordination materials by using Ag, instead of the common transition metals (Fe, Al, Cu) or the other noble metals (Rh and Ru) used to date^12^. Additionally, this suggests the possibility of using other monovalent metals such as K^+^ or Na^+^ to form functional phenolic coatings, as these have been used along with Ag^+^ to form other coordination materials such as metal–organic frameworks. Moreover, the broad-spectrum antimicrobial protection of the Ag/TA coatings against viruses, bacteria, and fungi offers a new route for integrating silver with textiles for long-term use and is amenable to both at-home (spraying) and industrial (immersive) application. Finally, TA and MPN coatings have previously been shown to stick to nearly any surface as TA can simultaneously demonstrate multiple interactions including electrostatics, hydrogen bonding, pi-pi stacking, and hydrophobic interactions^11,41,42^, and we therefore foresee that this facile, rapid, and relatively sustainable approach will find use in various medical, health, and lifestyle industries.

## Supplementary Information

Supplementary Figures.
