# Supplementary material for: Rapid assembly of colorless antimicrobial and anti-odor coatings from polyphenols and silver
Source: Sci Rep. 2022 Feb 8;12:2071. doi: 10.1038/s41598-022-05553-9 (PMC8826873; doi:10.1038/s41598-022-05553-9)
Supplement: Supplementary file 1 — Supplementary Figures. [file 41598_2022_5553_MOESM1_ESM.pdf]

Supporting Information

Rapid Assembly of Colorless Antimicrobial and Anti-Odor Coatings from Polyphenols and Silver

Joseph J. Richardson,[a*] Wenting Liao,[a] Jincai Li,[a] Bohan Cheng,[a] Chenyu Wang,[a] Taku Maruyama,[a] Blaise L. Tardy,[b] Junling Guo,[c,d] Lingyun Zhao,[e] Wanping Aw,[f] Hirotaka Ejima[a,*]

a Department of Materials Engineering, School of Engineering, University of Tokyo, Tokyo 113−8656, Japan.

b Department of Bioproducts and Biosystems, School of Chemical Engineering, Aalto University, Espoo 02150, Finland.

c BMI Center for Biomass Materials and Nanointerfaces, College of Biomass Science and Engineering, Sichuan University, Chengdu, Sichuan 610065, China.

d State Key Laboratory of Polymer Materials Engineering, Sichuan University, Chengdu, Sichuan 610065, China.

e State Key Laboratory of New Ceramics and Fine Processing, School of Materials Science and Engineering, Tsinghua University, Beijing 100084, China

f Institute for Advanced Biosciences, Keio University, 246-2 Mizukami, Kakuganji, Tsuruoka, Yamagata, 997-0052, Japan

*Direct correspondence to: ejima@g.ecc.u-tokyo.ac.jp, richardson@g.ecc.u-tokyo.ac.jp

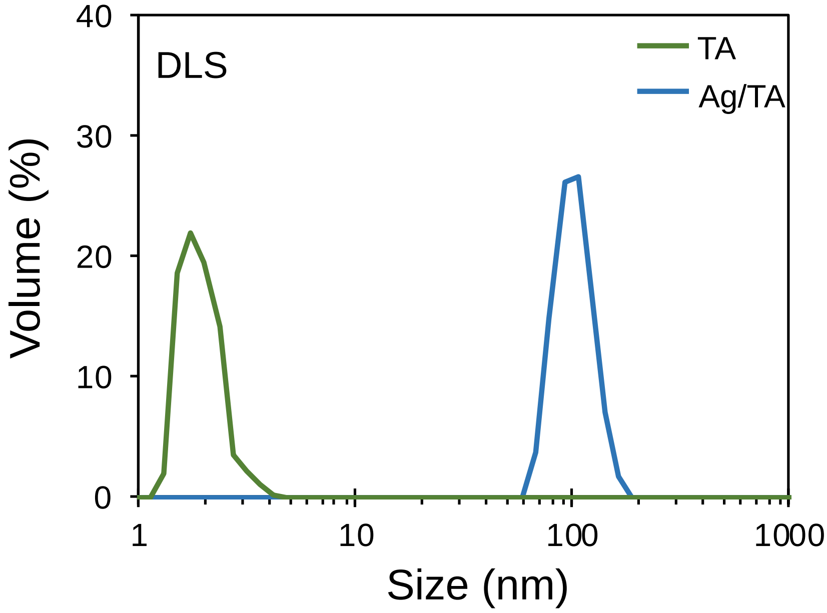

Figure S1. Dynamic light scattering results of aqueous solutions of TA and Ag/TA, where size refers to the diameter.

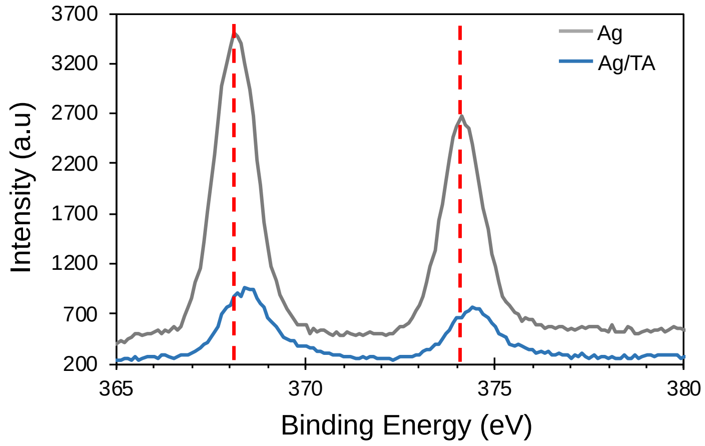

Figure S2. XPS spectra of $AgNO_3$ and Ag/TA complexes deposited on silicon wafers. The red dotted lines highlight the peaks of $AgNO_3$, where the Ag/TA peaks have clearly shifted.

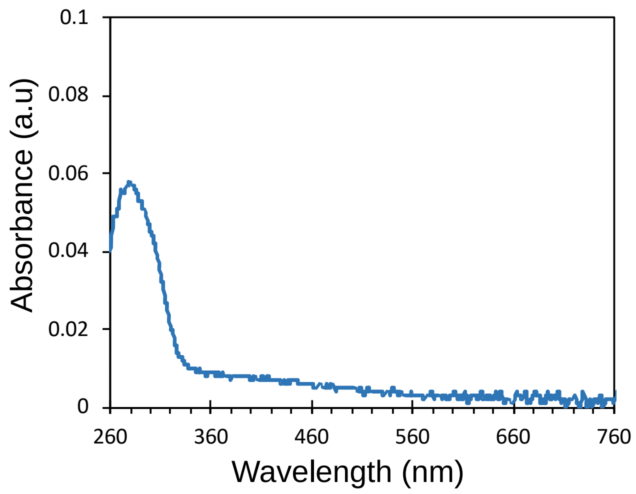

Figure S3. UV-Vis spectra of an Ag/TA-coated plastic cuvette after deposition for 2 h, with the signal normalized against an uncoated cuvette, demonstrating negligible absorbance in the visible spectrum even after prolonged assembly times.

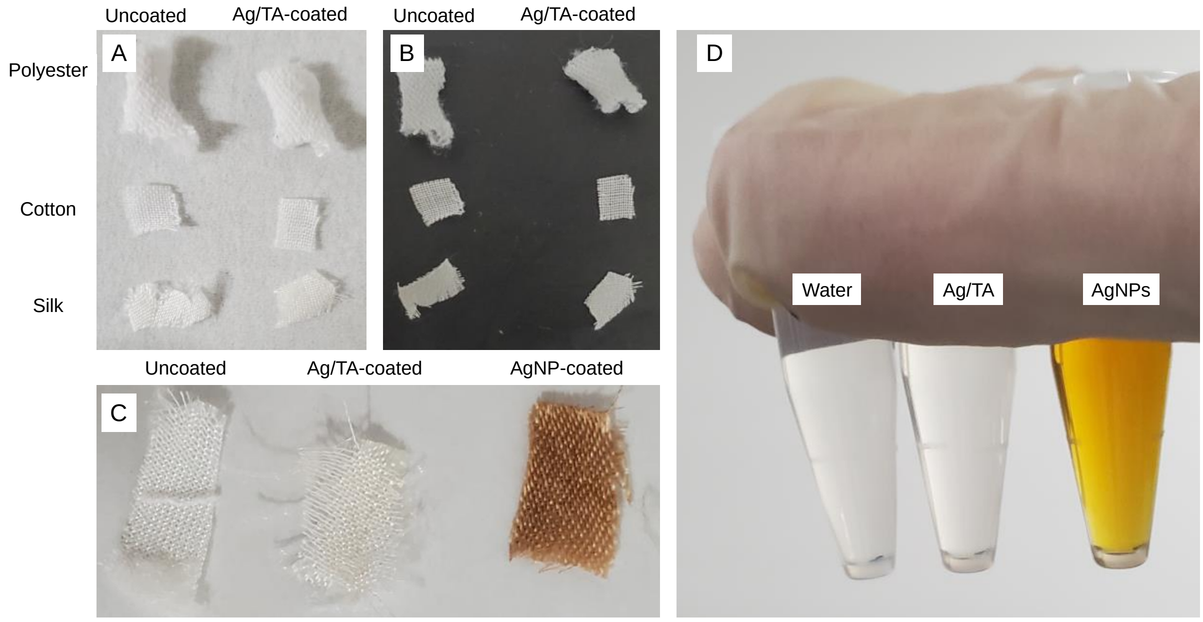

Figure S4. Images of uncoated and coated fabrics. A & B, images of the same samples just on different color backgrounds (A-white, B-black). C, images comparing Ag/TA-coated fabric to uncoated fabric and fabric coated with Ag nanoparticles (AgNPs). D, images of different solutions, highlighting the transparency and lack of visible light adsorption of Ag/TA complexes.

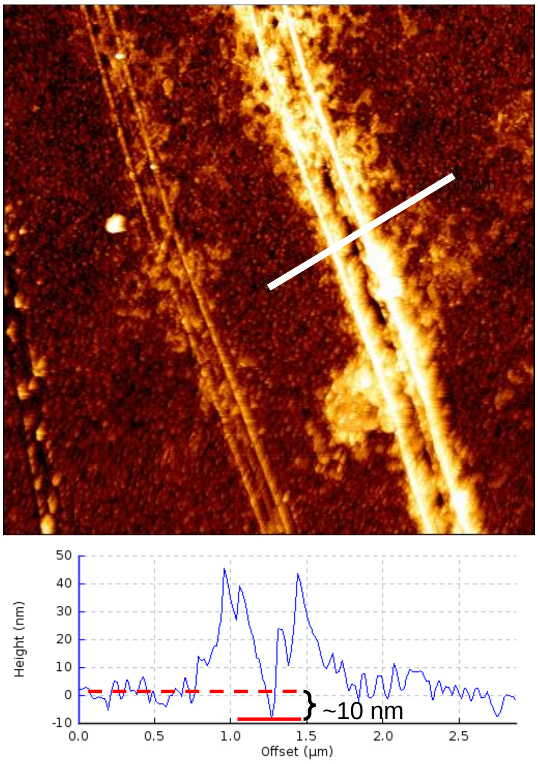

Figure S5. AFM image and corresponding height profile of an Ag/TA coating assembled over 20 min on a silicon wafer after scratching.

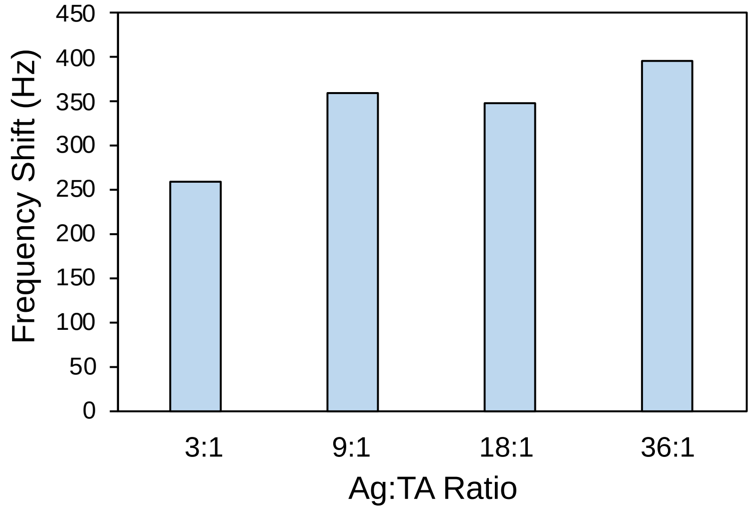

Figure S6. Mass deposited, as associated with frequency shift, on gold QCM chips after 1 min assembly time of different Ag to TA ratios, demonstrating the saturation of free binding site in TA.

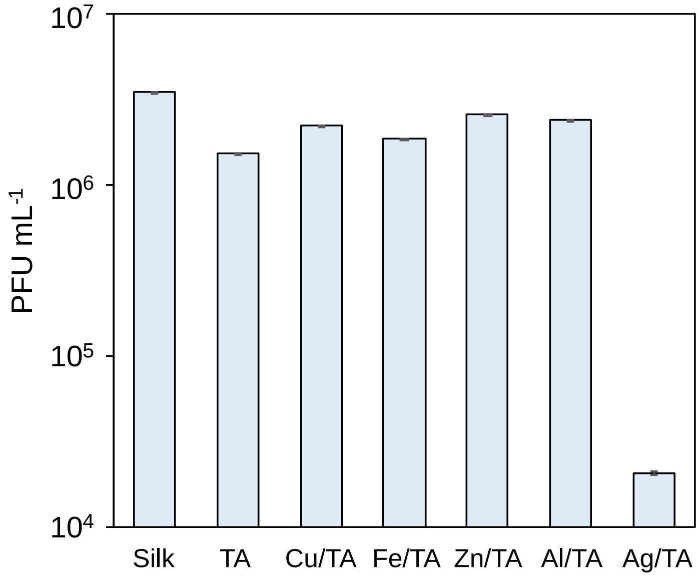

Figure S7. Amount of active virus recovered from bare and coated silk substrates after 20 min incubation followed by detachment from the substrate using surfactant.

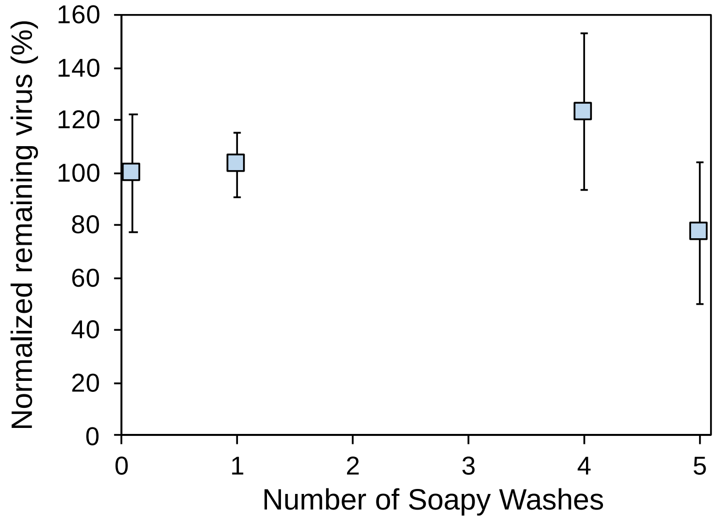

Figure S8. Resistance to washing of the antiviral properties of Ag/TA coatings comparing the first incubation to 1st, 4th, and 5th washes of the same coated silk substrate.

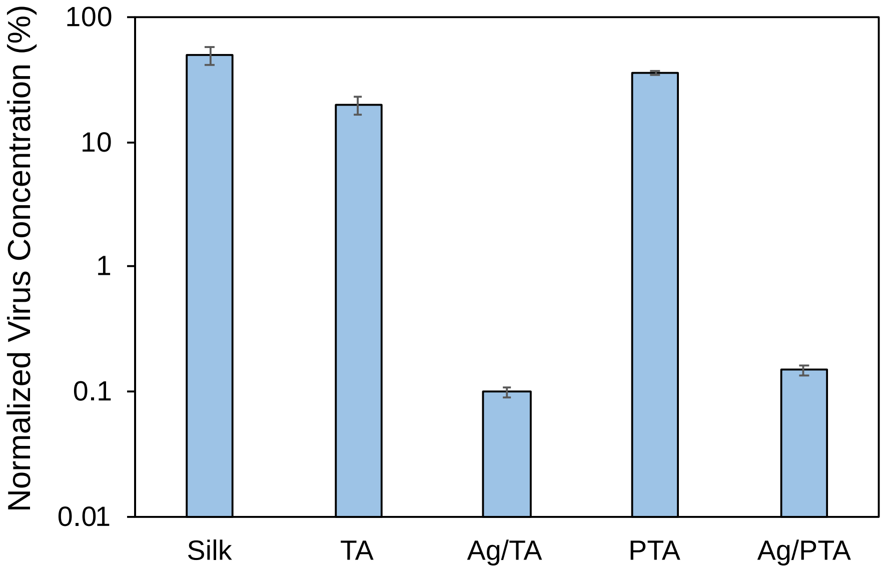

Figure S9. Antiviral potential of persimmon tannin (PTA) and Ag/PTA films in comparison to bare, TA-coated, and Ag/TA-coated silk, highlighting that different natural ligands can be used while still inhibiting viruses.

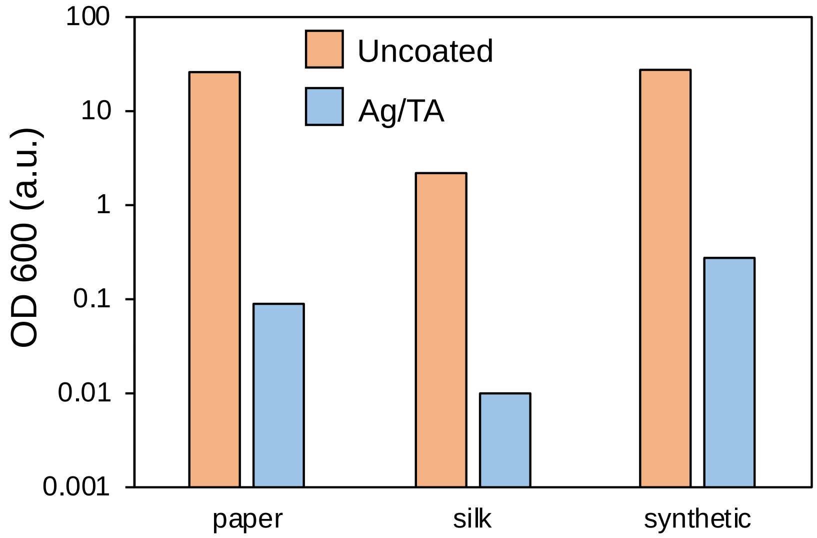

Figure S10. UV-Vis spectroscopy at 600 nm to determine the optical density (OD600) of uncoated and Ag/TA-coated fabric after incubation with *E. coli* on an agar plate, highlighting the antibacterial properties of the Ag/TA coatings.

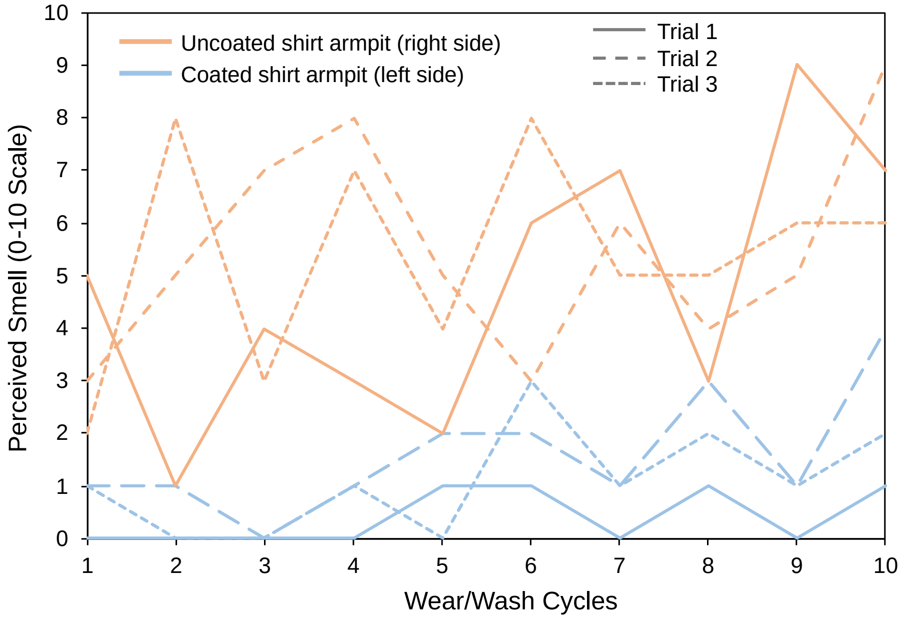

Figure S11. Raw data from the anti-odor experiments allowing for the direct comparison between the individual trials, highlighting that the coated armpit always had less smell.

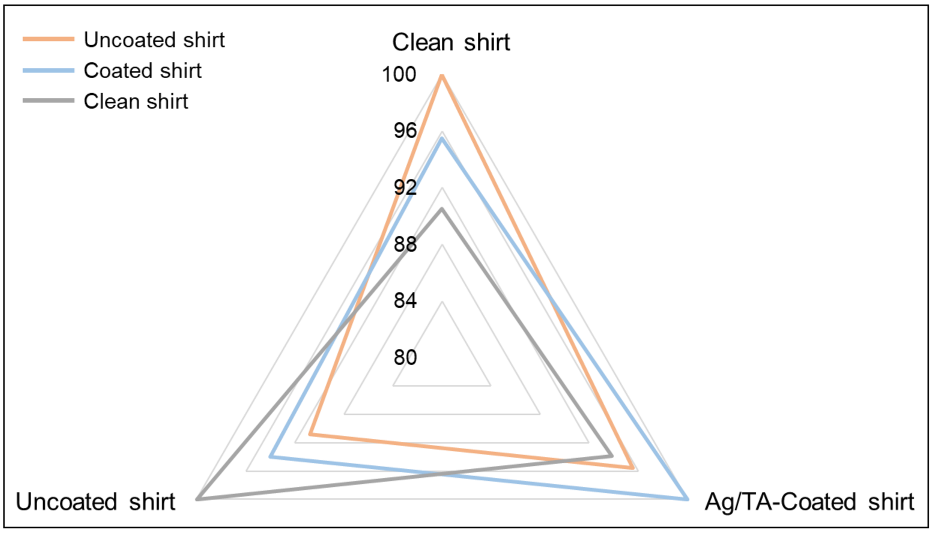

Figure S12. Similarity profile of the different samples measured by olfactometry highlighting that the clean and Ag/TA-coated samples had more similarities to each other than to the uncoated sample.

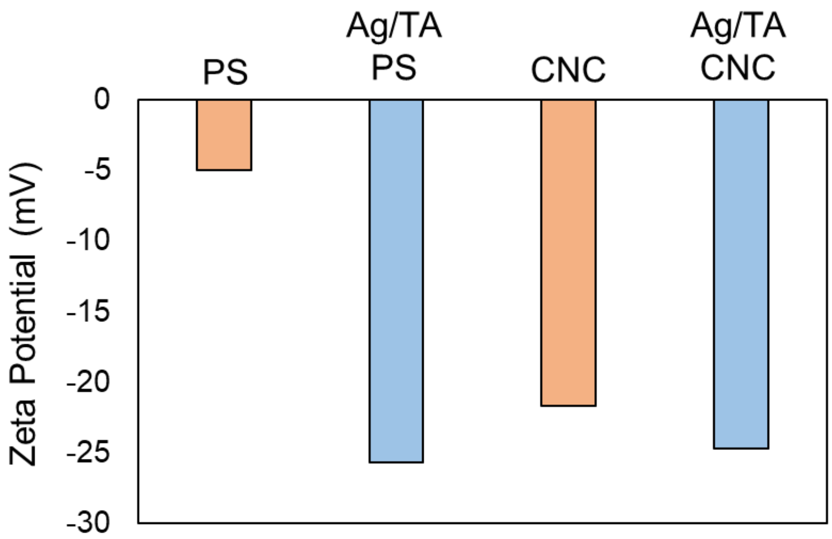

Figure S13. Zeta potential of polystyrene beads (PS) and cellulose nanocrystals (CNC) before and after Ag/TA coating.
